# Serological Remission Defined by Anti-Integrin αvβ6 Antibody Predicts Long-Term Clinical Remission of Ulcerative Colitis

**DOI:** 10.1016/j.gastha.2026.101043

**Published:** 2026-07-02

**Authors:** Noriaki Oguri, Jun Miyoshi, Nobuki Nemoto, Haruka Kamatsu, Haruka Wada, Takeshi Fujima, Ryota Ogihara, Noritaka Hibi, Hiromu Morikubo, Tatsuya Mitsui, Daisuke Saito, Mari Hayashida, Teppei Omori, Minoru Matsuura, Tadakazu Hisamatsu

**Affiliations:** 1 Department of Gastroenterology and Hepatology, Kyorin University School of Medicine, Tokyo, Japan; 2 Department of Gastroenterology and Hepatology, Kyorin University School of Medicine, Suginami Hospital, Tokyo, Japan

**Keywords:** Ulcerative Colitis, Integrin αvβ6, Biomarker, Clinical Remission, Clinical Relapse

## Abstract

**Background and Aims:**

Although management of ulcerative colitis (UC) has advanced with the treat-to-target strategy, biomarkers for relapse remain limited. The anti-integrin αvβ6 (V6) antibody reflects epithelial injury and could be a serological biomarker in UC. This retrospective cohort study evaluated whether the V6 antibody can predict relapse and be a serological target for sustained clinical remission.

**Methods:**

We measured V6 antibody levels in serum samples from patients with UC in clinical remission who had ≥3 years of follow-up. Relapse was defined as the need for initiation or modification (dose adjustment or switching) of systemic corticosteroids or molecular targeted drugs, or surgical intervention, due to worsening of disease activity (Lichtiger index ≥4). The ability of the V6 antibody to predict relapse and the cutoff value were investigated. Longitudinal intraindividual stability of the antibody level was evaluated.

**Results:**

Fifty-three of 149 patients experienced relapse. Seven and 2 patients required hospitalization and colectomy, respectively. The 1-year, 2-year, and 3-year cumulative relapse rates were 12.1%, 22.2%, and 32.9%, respectively. V6 antibody levels were significantly higher in patients who relapsed. Receiver-operating characteristic curve analyses showed considerable predictive ability (area under the curve, 0.73) at 1 to 3 years. The optimal cutoff for predicting 1-year relapse was 11.99U/mL, while ≤4.23U/mL yielded the highest 3-year negative predictive value. Cumulative 1-year, 2-year, and 3-year relapse rates were 0.0%, 0.0%, and 8.8%, respectively, in patients with ≤4.23U/mL. V6 antibody levels remained stable intraindividually and decreased on molecular-targeted therapy.

**Conclusion:**

The V6 antibody predicted long-term clinical remission of UC. The definition of serological response (≤11.99U/mL) and remission (≤4.23U/mL) based on this antibody potentially serves as a noninvasive therapeutic target in UC.

## Graphical Abstract

**Figure:**
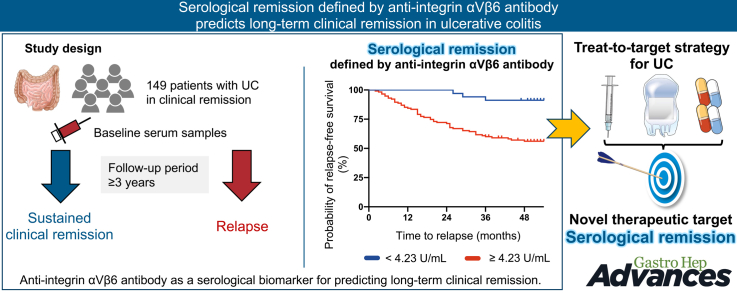

## Introduction

Ulcerative colitis (UC) is a major clinical phenotype of inflammatory bowel disease, typically affecting young adults, and is characterized by a relapsing–remitting disease course.^1^^,^^2^ Although the pathophysiology of UC remains incompletely understood, several mechanisms have been implicated, including disruption of intestinal barrier function, dysbiosis of the gut microbiota, and aberrant activation of the immune response.^3^^,^^4^ In recent years, the advent of molecular-targeted drug (MTD) therapy has enabled better symptom control and increased the likelihood of endoscopic remission.^5^^,^^6^ However, disease relapse remains a significant issue for many patients, and long-term disease management strategies are still required.

The treat-to-target (T2T) strategy, a therapeutic concept that optimizes therapy based on predefined treatment targets to achieve favorable therapeutic goals, is now widely accepted in UC.^7^ Implementation of the T2T strategy is considered associated with improved long-term outcomes, including sustained remission and improved quality of life.^8^^,^^9^ In UC, the short-term to medium-term targets of the T2T strategy include clinical and biological remission, with endoscopic remission being considered a long-term target. Furthermore, the STRIDE-II, a consensus framework, proposes achievement of histologic remission as an additional goal for better long-term outcomes.^7^ Biological remission of UC is assessed using biomarkers that serve as surrogate markers of disease activity, such as C-reactive protein and leucine-rich alpha-2 glycoprotein in blood, as well as fecal calprotectin and urinary prostaglandin E-major urinary metabolite.^7^^,^^10^^,^^11^ However, these biomarkers have limitations in terms of reflecting actual inflammatory activity and predicting the prognosis.^12^ Therefore, there is an unmet need for more accurate noninvasive biomarkers for the assessment of disease activity and prediction of the prognosis in patients with UC.

Increasing attention is now focused on the anti-integrin αvβ6 (V6) antibody detected in the serum of patients with UC.^13^, ^14^, ^15^, ^16^, ^17^, ^18^, ^19^ Integrin αvβ6, a heterodimer composed of αv and β6 subunits, is known to be expressed on the surface of intestinal epithelial cells.^13^ It interacts with the extracellular matrix and plays an important role in maintaining the integrity of the epithelial barrier.^20^^,^^21^ Serum V6 antibody levels have been reported to be higher in patients with UC than in healthy controls and patients with Crohn’s disease and to demonstrate high diagnostic performance.^13^^,^^15^ Moreover, increased V6 antibody levels have been observed even before clinical diagnosis of UC, suggesting that this antibody has potential value as a predictive biomarker for onset of the disease.^17^ Recent studies have also suggested associations between V6 antibody levels and disease activity in patients with UC.^15^, ^16^, ^17^ Other studies have highlighted plasmablast-skewed responses as a central feature in the pathogenesis of UC.^22^^,^^23^ Given that the V6 antibody is considered to be produced by pathogenic IgG plasmablasts, it could serve as a biomarker that more directly reflects underlying pathological mechanisms. However, the clinical significance of measuring serum V6 antibody levels within the framework of the T2T strategy has not yet been fully established. Therefore, the application of this biomarker in routine management of UC represents an important unmet clinical need.

In this study, given that maintaining remission is one of the key clinical challenges in the treatment of UC, we aimed to determine whether measurement of serum V6 antibody levels in patients with UC in clinical remission could help to predict subsequent disease relapse.

## Materials and Methods

### Study Subjects

The study retrospectively included patients with UC who were in clinical remission (Lichtiger index ≤3) and had serum samples stored at −80 °C after blood collection performed between March 2021 and November 2021 at Kyorin University Hospital in Japan. Eligible patients were required to have at least 3 years of follow-up data available after the time of baseline blood sampling. The diagnosis of UC was established based on clinical, endoscopic, and histopathological findings according to the diagnostic criteria for UC outlined in the current Japanese guideline for inflammatory bowel disease.^1^ Patients with a history of intestinal resection were excluded. Relapse was defined as the need for initiation or modification (dose adjustment or switching) of systemic corticosteroids or MTD, or surgical intervention, due to worsening of disease activity (Lichtiger index ≥4).

### Collection of Samples and Data

After blood sampling, centrifugation was performed immediately at 3500 rpm for 8 min. The supernatant was then collected as serum and stored at −80 °C until analysis. Frozen serum samples obtained at multiple time points were used for longitudinal analysis of antibody levels. Samples available from previous studies (clinical approval numbers 1378 and 1714), including baseline and follow-up time points (T1 [1.5–4.5 months], T2 [4.5–7.5 months], T3 [7.5–10.5 months], and T4 [10.5–13.5 months]), as well as those collected during active disease, were included in this analysis. Clinical data, including Lichtiger index, were systematically assessed and recorded at each outpatient visit as part of routine clinical practice. Patients were also instructed to contact the hospital in the event of symptom exacerbation, allowing additional assessments to be performed as needed. Information on medication use, including initiation and modification of therapy, was comprehensively captured through electronic medical records.

### Enzyme-Linked Immunosorbent Assay for Anti-Integrin αVβ6 Antibody

Serum V6 antibody levels were measured using a commercially available V6 enzyme-linked immunosorbent assay kit (MBL, Minato-ku, Tokyo, Japan) according to the manufacturer’s protocol.^15^ Briefly, 5 μL of serum were diluted 1:101 in reaction buffer and incubated on a plate coated with integrin αVβ6 for the primary antigen–antibody reaction. After washing, labeled antibody solution was added for the secondary antibody–enzyme reaction. Following further washing, an enzymatic color reaction was performed and stopped by addition of stop solution. Absorbance was measured at 450 nm (reference wavelength 620 nm) using a Varioskan instrument (Thermo Fisher Scientific, Waltham, MA). V6 antibody levels were calculated based on a standard curve generated from known levels using a four-parameter logistic regression model.

### Statistical Analysis

For comparisons between groups, the Mann–Whitney *U* test was used for unpaired continuous variables, the Wilcoxon signed-rank test for paired data, and Fisher exact test for categorical variables. To evaluate the discriminative ability of serum V6 antibody levels for predicting relapse, receiver-operating characteristic (ROC) curves were constructed using relapse and remission data at 1, 2, and 3 years, and the area under the curve (AUC) was calculated. The optimal cutoff value was determined as the point maximizing the Youden index. Cumulative relapse-free survival was analyzed using the Kaplan–Meier method, and group differences were examined using the log-rank test. The correlation between the V6 antibody level and time to relapse was evaluated using Spearman rank correlation coefficient. The intraclass correlation coefficient (ICC) was calculated for assessment of intraindividual consistency of antibody levels over time. A linear mixed-effects model was constructed using the lme4 package (https://cran.r-project.org/web/packages/lme4/index.html) in R, treating subjects as random effects. This approach accounted for inter-individual variability and allowed analysis of longitudinal data with missing values using restricted maximum likelihood estimation. All statistical analyses were performed using R software (version 4.1.3; https://www.r-project.org/) and GraphPad Prism (version 8.4.3; GraphPad Software Inc, San Diego, CA). A two-sided *P* value of <.05 was considered statistically significant.

## Results

### Patient Characteristics

A total of 149 patients with UC who were in clinical remission at the time of serum sampling and followed for more than 3 years were included in the analysis. The patient demographic and clinical characteristics are summarized in Table 1. The median age was 40 years (interquartile range [IQR] 28, 52), and 96 (64.4%) of the patients were male. The disease extent was pancolitis in 107 patients (71.8%), left-sided colitis in 2 (26.9%), and proctitis in 40 (1.3%). MTD therapy was used in 101 patients (67.8%), azathioprine in 54 (36.2%), and 6-mercaptopurine in 9 (6.0%). The median observation period was 52 months (IQR 52, 53). The median duration of disease from diagnosis to the baseline blood sampling was 87 months (IQR 54.25, 172.3), and the median interval between the most recent remission induction therapy for UC and the baseline blood sampling was 23 months (IQR 11, 48).

**Table 1:** Patient Demographic and Clinical Characteristics

Characteristics	n = 149
Age, y	40 [28, 52]
Sex	
Male	96 (64.43%)
Female	53 (35.57%)
Observation period, mo	52 [52, 53]
Disease type, pancolitis/left-sided colitis/proctitis	107/40/2 (71.81%/26.85%/1.34%)
Naïve to molecular-targeted therapy	48 (32.21%)
Disease duration, mo (n = 146)	87 [54.25, 172.3]
Time since last induction therapy, mo	23 [11, 48]
Lichtiger index	1 [0, 2]
Albumin, g/dL (n = 148)	4.20 [4.00, 4.40]
C-reactive protein, mg/dL (n = 147)	0.05 [0.02, 0.14]
Baseline treatment	
5-aminosalicylic acid	116 (77.85%)
Salazosulfapyridine	7 (4.70%)
Oral corticosteroids	3 (2.01%)
Azathioprine	54 (36.24%)
6-mercaptopurine	9 (6.04%)
Infliximab	33 (22.15%)
Adalimumab	12 (8.05%)
Golimumab	14 (9.40%)
Vedolizumab	25 (16.78%)
Ustekinumab	5 (3.36%)
Tofacitinib	7 (4.70%)
Upadacitinib	1 (0.67%)
Risankizumab	1 (0.67%)
Mirikizumab	3 (2.01%)
Probiotics	107 (71.81%)

Data are presented as the number (percentage) or as the median (interquartile range).

### High Serum Anti-Integrin αvβ6 Antibody Levels Predict Relapse in Patients With Ulcerative Colitis in Clinical Remission

**Figure 1:**
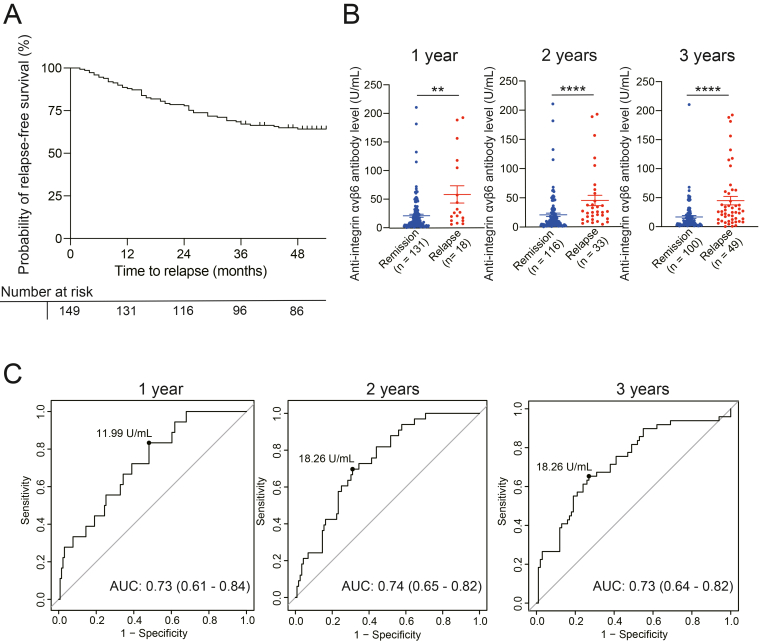
Ability of serum anti-integrin αvβ6 antibody levels to predict relapse in patients with ulcerative colitis. (A) Kaplan–Meier curve showing cumulative relapse rates in the overall cohort. (B) Comparisons of anti-integrin αvβ6 (V6) antibody concentrations between patients who relapsed and those who remained in remission at 1, 2, and 3 years (1 year, 58.35 ± 15.11 U/mL vs 21.62 ± 2.68 U/mL, *P* = .0015; 2 years, 44.16 U/mL ± 8.59 vs 20.67 ± 2.95 U/mL, *P* < .0001; 3 years, 45.01 ± 8.81 U/mL vs 16.85 ± 2.50 U/mL, *P* < .0001). (C) Receiver-operating characteristic (ROC) curves demonstrating the prediction performance of V6 antibody concentrations for relapse at 1, 2, and 3 years. Data are presented as the mean ± standard error of the mean. ∗∗*P* < .01, ∗∗∗∗*P* < .0001, Mann–Whitney *U* test (two-sided) for comparisons of 2 groups. The optimal cutoff value for each ROC curve was determined as the point maximizing the Youden index.

The cumulative relapse rate during the observation period is shown by the Kaplan–Meier curve in Figure 1A. Relapse occurred in 53 patients, with a median time to relapse of 17 months (IQR 9.5, 29). Two patients (1.3%) underwent colectomy during the observation period, all of which occurred following initiation or modification (dose adjustment or switching) of systemic corticosteroids or MTD. Seven patients with clinical relapse required hospitalization related to treatment. All patients who required systemic corticosteroids, initiated an MTD, switched MTDs, or underwent dose intensification of an MTD had a Lichtiger index ≥4 at that time. The cumulative relapse rates at 1, 2, and 3 years were 12.1%, 22.2%, and 32.9%, respectively. For all time points, baseline V6 antibody levels were significantly higher in patients who experienced relapse than in those who maintained clinical remission (1 year, 58.35 ± 15.11 U/mL vs 21.62 ± 2.68 U/mL, *P* = .0015; 2 years, 44.16 ± 8.59 U/mL vs 20.67 ± 2.95 U/mL, *P* < .0001; 3 years, 45.01 ± 8.81 U/mL vs 16.85 ± 2.50 U/mL, *P* < .0001) (Figure 1B).

**Figure 2:**
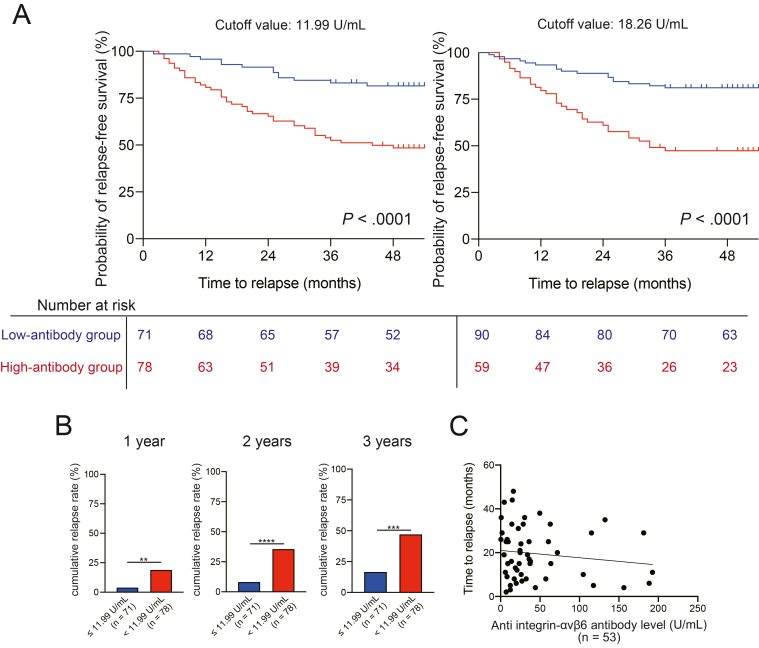
Stratification by the serum anti-integrin αvβ6 antibody level and relapse-free survival. (A) Kaplan–Meier curves showing relapse-free survival according to the optimal cutoff values (11.99 U/mL, *P* < .0001; 18.26 U/mL, *P* = .0001). (B) Cumulative relapse rates in patients stratified by the cutoff value of 11.99 U/mL (1 year, 4.2% vs 19.2%, *P* = .0054; 2 years, 8.5% vs 35.9%, *P* < .0001; 3 years, 16.9% vs 47.4%, *P* = .0001). (C) Correlation between anti-integrin αvβ6 antibody level and time to relapse (*P* = .3185, *R*^2^ = 0.0195). ∗∗*P* < .01, ∗∗∗*P* < .001, ∗∗∗∗*P* < .0001, log-rank test for comparisons of Kaplan–Meier curves; Fisher exact test (two-sided) for comparison of categorical variables between 2 groups; Spearman rank correlation coefficient for correlation analysis.

ROC curve analyses demonstrated considerable prediction performance across the time points, with AUC of 0.73 (95% confidence interval [CI] 0.61 to 0.84) at 1 year, 0.74 (95% CI 0.65–0.82) at 2 years, and 0.73 (95% CI 0.64–0.82) at 3 years (Figure 1C, Table 2). The optimal cutoff values for prediction of relapse were 11.99 U/mL at 1 year and 18.26 U/mL at both 2 and 3 years. Kaplan–Meier analyses based on these thresholds revealed significantly higher cumulative relapse rates in the high-antibody groups at both cutoff values (11.99 U/mL, *P* < .0001; 18.26 U/mL, *P* < .0001) (Figure 2A).

**Table 2:** Optimal Anti-Integrin αvβ6 Antibody Cutoff Levels That Predicted Relapse Over 3 Y

Years	Cutoff value (U/mL)	Sensitivity (%)	Specificity (%)	PPV (%)	NPV (%)	AUC (95% CI)
1 y	11.99	83.33	51.91	19.23	95.77	0.73 (0.61–0.84)
2 y	18.26	69.70	68.97	38.98	88.89	0.74 (0.65–0.83)
3 y	18.26	65.31	73.00	54.24	81.11	0.73 (0.64–0.82)

AUC, area under the curve; CI, confidence interval; NPV, negative predictive value; PPV, positive predictive value.

The finding that the optimal cutoff value obtained at 1 year (11.99 U/mL) was lower than that at 2 and 3 years (18.26 U/mL) suggests that the 11.99 U/mL threshold indicates a risk of earlier relapse and may be more clinically useful than the 18.26 U/mL threshold for predicting relapse in patients with UC. Using the threshold of 11.99 U/mL, the cumulative relapse rates were significantly higher in the high-antibody group than in the low-antibody group at 1, 2, and 3 years (1 year, 19.2% vs 4.2%, *P* = .0054; 2 years, 35.9% vs 8.5%, *P* < .0001; 3 years, 47.4% vs 16.9%, *P* = .0001) (Figure 2B). Three of 71 patients with a V6 antibody level of ≤11.99 U/mL required hospitalization and none required colectomy during 3 years of follow-up. No significant correlation was observed between the V6 antibody level and time to relapse (*P* = .3185, *R*^2^ = 0.0195) (Figure 2C).

### A Low Anti-Integrin αvβ6 Antibody Level as a Potential Serological Target for Achieving Sustained Clinical Remission

**Figure 3:**
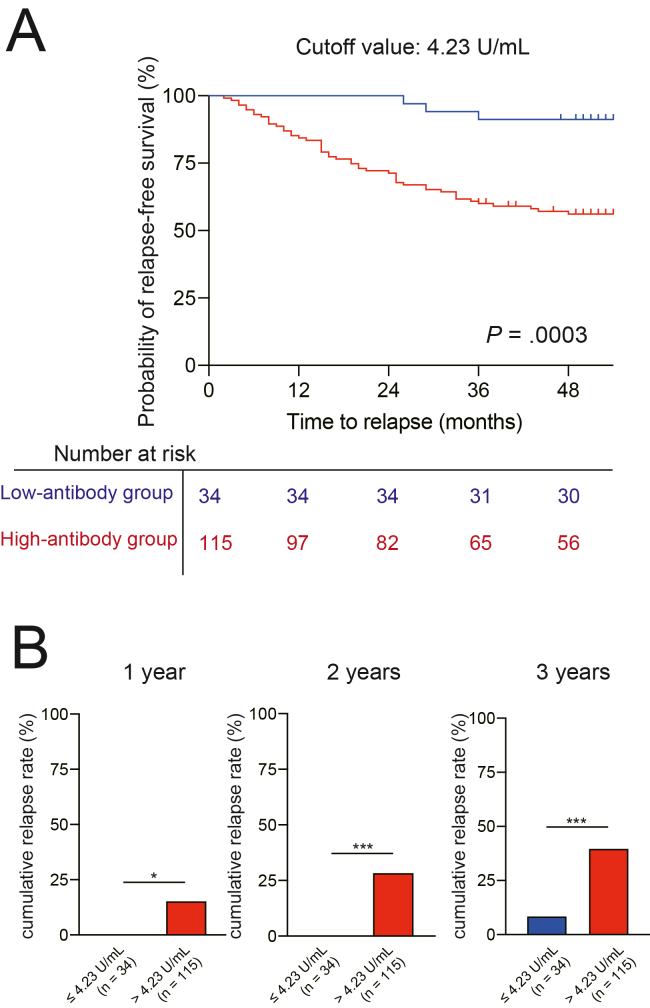
Ability of a low serum anti-integrin αvβ6 antibody level to predict sustained clinical remission. (A) Kaplan–Meier curve showing relapse-free survival according to the cutoff of 4.23 U/mL with the highest negative predictive value (NPV) for 3-year relapse (*P* = .0003). (B) Cumulative relapse rates in patients stratified by the cutoff of 4.23 U/mL (1 year, 0.0% vs 15.65%, *P* = .0133; 2 years, 0.00% vs 28.70%, *P* = .0001; 3 years, 8.82% vs 40.00%, *P* = .0004). ∗*P* < .05, ∗∗*P* < .01, ∗∗∗*P* < .001, log-rank test for comparison of Kaplan–Meier curves; Fisher exact test (two-sided) for comparison of categorical variables between 2 groups.

Next, we examined whether the V6 antibody level could serve as a therapeutic target for achieving long-term clinical remission. The cutoff value associated with the highest negative predictive value of 91.2% for relapse within 3 years was 4.23 U/mL. The low-antibody group (≤4.23 U/mL; n = 34) had significantly lower cumulative relapse rates over time (*P* = .0003) (Figure 3A). The cumulative relapse rates in the low-antibody group at 1, 2, and 3 years were 0.0%, 0.0%, and 8.8%, respectively, indicating that more than 90% of patients maintained clinical remission for longer than 3 years. In contrast, in the high-antibody group (>4.23 U/mL; n = 115), cumulative relapse rates were 15.7%, 28.7%, and 40.0% at 1, 2, and 3 years, respectively (1 year, *P* = .0133; 2 years, *P* = .0001; 3 years, *P* = .0004) (Figure 3B). None of the 34 patients with a V6 antibody level of ≤4.23 U/mL required hospitalization or colectomy during 3 years of follow-up.

### Intraindividual Stability of Anti-Integrin αvβ6 Antibody Levels

**Figure 4:**
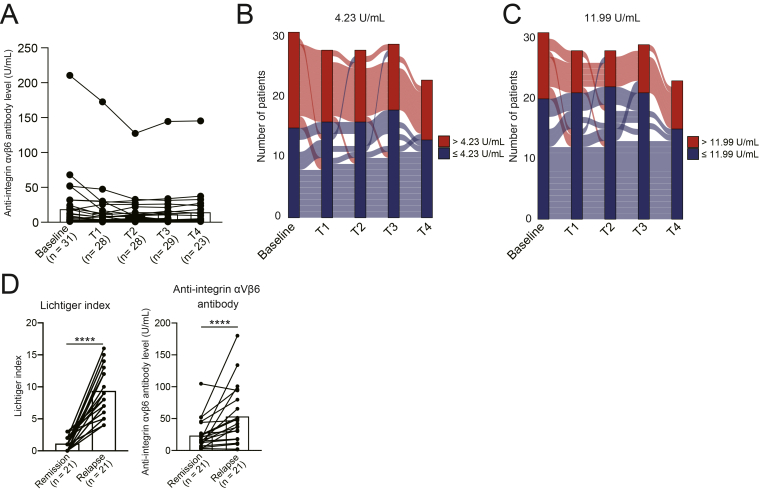
Stability of serum anti-integrin αvβ6 antibody levels in individuals during clinical remission. (A) Longitudinal analysis showing anti-integrin αvβ6 (V6) antibody concentrations in patients who maintained remission without a change in treatment. (B, C) Alluvial plots across serial samples using the cutoff values of 4.23 U/mL and 11.99 U/mL. (D) Comparison of Lichtiger index values and V6 antibody levels between patients with remission and those with active disease in paired samples. ∗*P* < .05, ∗∗*P* < .01, ∗∗∗∗*P* < .0001, pairwise comparisons (two-sided) were performed using estimated marginal means, accounting for repeated measures, and adjusted using the Benjamini–Hochberg method for longitudinal analysis. The Wilcoxon signed-rank test (two-sided) was performed for comparisons of paired data.

For clinical application as a biomarker or therapeutic target, the V6 antibody level should remain stable over time in patients maintaining clinical remission without changes in treatment. The intraindividual stability of the V6 antibody level was analyzed in 31 patients who remained in clinical remission throughout the observation period and had longitudinal serum samples available. Samples were collected at baseline and at 4 subsequent time points: T1 (1.5–4.5 months), T2 (4.5–7.5 months), T3 (7.5–10.5 months), and T4 (10.5–13.5 months). There were no changes in MTD or corticosteroid therapy in any of these patients during this period. Using a linear mixed-effects model accounting for repeated measures, no significant temporal change in the V6 antibody level was detected in any of these patients (Figure 4A). The ICC for intraindividual measurements was high (adjusted ICC, 0.92), indicating excellent stability. When patients were stratified using the strict cutoff of 4.23 U/mL for nonrelapse within 3 years, classification at baseline was consistent across subsequent time points (Figure 4B). Similarly, stratification by the cutoff of 11.99 U/mL showed stable group allocation over time (Figure 4B).

In contrast, in 21 patients with paired samples during clinical remission (median Lichtiger index = 1 [IQR 0, 2]) and active disease (median Lichtiger index = 9 [IQR 6.5, 12.5]), V6 antibody levels were significantly higher during active disease (24.14 ± 5.32 U/mL vs 54.12 ± 10.03 U/mL; *P* < .0001) (Figure 4C).

### Lowering the Anti-Integrin αvβ6 Antibody Level as a Potential Therapeutic Approach Leading to Long-Term Clinical Remission

**Figure 5:**
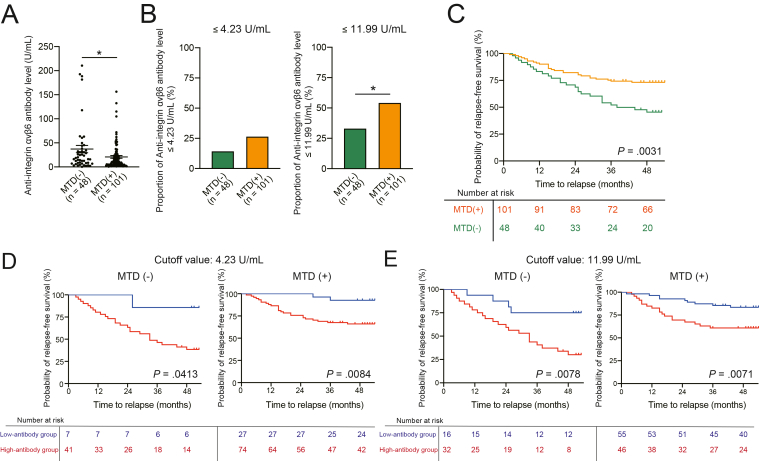
Association between molecular-targeted therapy and serum anti-integrin αvβ6 antibody levels. (A) Comparison of anti-integrin αvβ6 antibody levels between patients without and with use of molecular-targeted drug (MTD) therapy (37.09 ± 7.55 U/mL vs 20.82 ± 2.74 U/mL, *P* = .0250). (B) Proportion of patients achieving an anti-integrin αvβ6 antibody level of ≤4.23 U/mL or ≤11.99 U/mL without or with use of MTD therapy (≤4.23 U/mL, 14.58% vs 26.73%, *P* = .1428; ≤11.99 U/mL, 33.33% vs 54.46%, *P* = .0221). (C) Kaplan–Meier curves showing relapse-free survival stratified by MTD use (*P* = .0031). (D) Kaplan–Meier curves showing relapse-free survival stratified by the cutoff of 4.23 U/mL according to MTD use [MTD(−) group, *P* = .0413; MTD(+) group, *P* = .0084]. (E) Kaplan–Meier curves showing relapse-free survival stratified by the cutoff of 11.99 U/mL according to MTD use [MTD(−) group, *P* = .0078; MTD(+) group, *P* = .0071]. Data are presented as the mean ± standard error of the mean. ∗∗*P* < .01, ∗∗∗∗*P* < .0001, Mann–Whitney *U* test (two-sided) for comparisons of 2 groups; Fisher exact test (two-sided) for categorical variables between 2 groups; log-rank test for comparison of Kaplan–Meier curves.

The mean V6 antibody level was significantly lower in patients who received MTD therapy [the MTD(+) group] than in those who did not [the MTD(−) group] (37.09 ± 7.55 U/mL vs 20.82 ± 2.74 U/mL, *P* = .0250) (Figure 5A). The proportion of patients who achieved a V6 antibody level <4.23 U/mL was higher in the MTD(+) group than in the MTD(−) group (14.6% vs 26.7%, *P* = .1428) (Figure 5B), although the difference was not statistically significant Meanwhile, the proportion of patients who achieved a V6 antibody level below the 11.99 U/mL threshold was significantly greater in the MTD(+) group than in the MTD(−) group (33.3% vs 54.5%, *P* = .0221) (Figure 5B).

The cumulative relapse rate was significantly lower in the MTD(+) group than in the MTD(−) group (*P* = .0031) (Figure 5C). When stratified by a V6 antibody cutoff value of 4.23 U/mL, lower relapse rates were observed in the low-antibody group (≤4.23 U/mL) regardless of MTD use [MTD(−), *P* = .0413; MTD(+), *P* = .0084] (Figure 5D). Similarly, stratification using the 11.99 U/mL cutoff demonstrated significantly lower relapse rates in the low-antibody group (≤11.99 U/mL) irrespective of MTD use [MTD(−), *P* = .0078, MTD(+), *P* = .0071] (Figure 5E). Detailed distributions of each MTD in the subgroups defined by the 4.23 U/mL and 11.99 U/mL cutoffs are provided in Supplementary Table 1.

## Discussion

This study demonstrated that the V6 antibody level has considerable predictive ability for clinical relapse in patients with UC in clinical remission. In particular, over 90% of patients with a V6 antibody level of ≤4.23 U/mL maintained clinical remission for more than 3 years. This study also showed the stability of antibody levels measured in patients with UC in clinical remission. Furthermore, the results may suggest that therapeutic interventions that lower the antibody level could contribute to maintaining long-term clinical remission. These findings indicate that the serum V6 antibody level is a useful biomarker for predicting relapse in patients with UC in clinical remission and could serve as a potential therapeutic target for long-term clinical remission.

ROC curve analyses using relapse rates at 1, 2, and 3 years showed consistently high predictive accuracy, with AUC of approximately 0.73 at all time points. The optimal cutoff value for prediction of 1-year relapse was 11.99 U/mL and that for 2-year and 3-year relapse was 18.26 U/mL. Considering that the higher cutoff might underestimate the risk of early relapse, we considered 11.99 U/mL to be a clinically reasonable threshold. Stratification based on this value revealed significant differences in cumulative relapse rates, confirming the potential of V6 antibody levels for prediction of relapse in patients with UC. However, given that no correlation was observed between the V6 antibody level and time to relapse, we considered that the V6 antibody level may serve better as a marker of long-term maintenance of clinical remission rather than as a predictor of relapse in the shorter term. On the basis of this concept, we identified 4.23 U/mL as the cutoff with the highest negative predictive value for relapse within 3 years and demonstrated that this cutoff had strong predictive ability for sustained remission.

Longitudinal analysis of intraindividual V6 antibody levels in patients who maintained clinical remission without changes in therapy revealed no significant temporal variation, with an ICC of 0.92, indicating high reproducibility. These findings suggest that the V6 antibody can be measured stably under consistent conditions, supporting its stability as a single-point biomarker for patients in clinical remission.

Interestingly, we observed that V6 antibody levels were significantly lower in patients who received MTD therapy than in those who did not. A causal relationship between MTD use and reduction in V6 antibody levels cannot be definitively established due to the observational nature of this study. However, considering that patients treated with MTDs likely represent cases which originally had severe activity and/or those refractory to conventional therapy, this finding suggests that MTD therapy may reduce the V6 antibody level by reducing mucosal inflammation. Given a previous report showing a correlation between V6 antibody levels and endoscopic activity,^24^ our results indicate that V6 antibody levels are modifiable by therapeutic intervention. However, regardless of MTD use, long-term clinical remission rates were significantly higher when the V6 antibody level was ≤4.23 U/mL than when it was >4.23 U/mL. Collectively, these findings suggest that the V6 antibody is a promising serological target in the T2T strategy for UC irrespective of the treatment regimen. Prospective studies that directly assess the impact of therapeutic interventions on the dynamics of V6 antibody levels are needed to validate the value of this antibody as a serological target in the T2T strategy.

Patients with V6 antibody levels ≤4.23 U/mL showed clinical remission maintenance rates of 100% at 1 and 2 years and 91.2% at 3 years, compared with 71.3% and 60.0%, respectively, in patients with levels above this threshold. However, only 22.8% of patients achieved a V6 antibody level ≤4.23 U/mL, suggesting that while this threshold identifies patients with excellent long-term outcomes, it may be difficult to achieve in routine clinical practice. In contrast, 47.7% of patients had a V6 antibody level ≤11.99 U/mL, and their 3-year clinical remission maintenance rate was 83.1%, compared with 52.6% among those above this level. Therefore, when incorporating the V6 antibody level into a T2T strategy as a serological biomarker, we propose defining ≤4.23 U/mL as a strict target corresponding to serological remission and ≤11.99 U/mL as a more feasible and practical target corresponding to a serological response. Such stepwise thresholds may be clinically useful in implementing a biomarker-based T2T strategy.

Integrin αvβ6 is known to be upregulated in epithelial cells during mucosal wound healing.^13^^,^^20^ A recent study demonstrated that plasmablasts in the intestinal mucosa of patients with UC are skewed from immunoglobulin A-producing toward immunoglobulin G (IgG)-producing subsets in comparison with healthy individuals. Moreover, IgG-producing plasmablasts in the colonic mucosa have been shown to generate the V6 antibody, suggesting their potential involvement in the pathogenesis of UC.^22^ Given that production of the V6 antibody appears to arise directly from these pathogenic IgG plasmablast responses, unlike other inflammation biomarkers (eg, C-reactive protein or fecal calprotectin), V6 antibody levels could serve as a biomarker that more closely reflects the underlying immunologic activity of UC.

We speculated that residual epithelial damage in UC in clinical remission without complete mucosal healing creates an environment that promotes antibody production. This notion is supported by a report showing a correlation between V6 antibody levels and endoscopic disease activity.^24^ It is well recognized that some patients in clinical remission lack complete endoscopic healing and that those achieving a Mayo endoscopic subscore of 0 have better long-term outcomes than those with a subscore of 1.^7^^,^^25^, ^26^, ^27^, ^28^ Therefore, stratification based on the V6 antibody level could reflect the degree of mucosal healing in patients with UC in clinical remission. That is, patients with lower V6 antibody levels during clinical remission could be in a state closer to mucosal healing, which could account for their favorable long-term outcomes.

This study has several limitations. First, it was a single-center analysis that used stored blood samples. Although based on real-world data from routine clinical practice, potential selection bias cannot be completely excluded. Second, we used an enzyme-linked immunosorbent kit intended for research purposes because measurement of the V6 antibody is not yet clinically standardized. Therefore, caution is needed when generalizing the measured values for clinical application. Third, the study evaluated baseline V6 antibody levels in relation to long-term clinical outcomes, but remission and relapse were defined clinically, without concurrent endoscopic or histologic assessment. Therefore, some patients classified as being in remission clinically might have had subclinical mucosal or histologic disease activity. Nevertheless, invasive endoscopic or histologic evaluations are often impractical in real-world settings. Given that clinical disease activity indices serve as helpful reference information in routine UC management, including in long-term follow-up settings, the present study can still be considered to provide clinically meaningful insights into the management of UC in real-world clinical practice. The investigation on other biomarkers (eg, fecal calprotectin) could also be considered an interesting clinical question. Future comparisons of serum V6 antibody levels with endoscopic and histologic findings, as well as with other biomarkers, could clarify whether this biomarker can serve as a noninvasive surrogate for mucosal inflammation. Furthermore, because baseline samples were obtained during established clinical remission rather than at the time of initial diagnosis, the optimal timing of sampling within the remission period remains unclear. Finally, our analysis focused on patients in sustained clinical remission; therefore, no longitudinal evaluation of changes in the V6 antibody level before and after specific therapeutic interventions was performed. Prospective multicenter studies are needed to validate the optimal cutoff values for clinical use and to investigate how different treatment strategies influence V6 antibody levels and outcomes.

## Conclusion

This study demonstrated that the serum V6 antibody is a promising serological target for long-term clinical remission in UC. The concept of serological remission and serological response defined by V6 antibody thresholds has the potential to provide a less invasive biomarker-based T2T strategy, contributing to the advancement of personalized treatment in UC.

## Supplementary Materials

Supplementary Table 1

Extended PDF
