# Supplementary material for: Serological Remission Defined by Anti-Integrin αvβ6 Antibody Predicts Long-Term Clinical Remission of Ulcerative Colitis
Source: Gastro Hep Adv. 2026 Jul 2;5(9):101043. doi: 10.1016/j.gastha.2026.101043 (PMC13446341; doi:10.1016/j.gastha.2026.101043)
Supplement: Supplementary file 1 [file mmc1.pdf]

Supplementary Table 1. Distribution of molecular targeted drugs in patients stratified by integrin-αVβ6 antibody levels.

	Cutoff 4.23 U/mL (n = 101)		Cutoff 11.99 U/mL (n = 101)	
	≤ 4.23 U/mL (n = 27)	> 4.23 U/mL (n = 74)	≤ 11.99 U/mL (n = 55)	> 11.99 U/mL (n = 46)
Infliximab	11 (40.7 %)	22 (29.7 %)	19 (34.5 %)	14 (30.4 %)
Adalimumab	3 (11.1 %)	9 (12.2 %)	9 (16.4 %)	3 (6.5 %)
Golimumab	2 (7.4 %)	12 (16.2 %)	3 (5.5 %)	11 (23.9 %)
Vedolizumab	5 (18.5 %)	20 (27.0 %)	13 (23.6 %)	12 (26.1 %)
Ustekinumab	3 (11.1 %)	2 (2.7 %)	4 (7.3 %)	1 (2.2 %)
Tofacitinib	2 (7.4 %)	5 (6.8 %)	4 (7.3 %)	3 (6.5 %)
Upadacitinib	0 (0%)	1 (1.4 %)	1 (1.8 %)	0 (0 %)
Risankizumab	0 (0%)	1 (1.4 %)	0 (0 %)	1 (2.2 %)
Mirikizumab	1 (3.7%)	2 (2.7 %)	2 (3.6 %)	1 (2.2 %)
